# Miscellaneous

**Published:** 1891-05

**Authors:** 


### MISCELLANEOUS.

### LYNCHED BY SPARROWS.

One day last Spring, as I was going down town I saw a flock of about fifty sparrows on the ground in a vacant lot on the corner of Thirteenth and Central streets. They were making as much fuss and noise as a political ward meeting, where every other man had a candidate for nomination, and all wanted to be heard at the same time. So intently were they engaged that I walked up to within twenty feet of them without their taking the alarm. I found that they had in their midst a full grown young rat who seemed to be completely cowed, and paralyzed with fear, and trembling all over. As long as he remained quiet they merely stood around and chattered and shrieked at him, but when he make a move they would pounce down in front of him and drive him back. There was an old wooden side-walk at the time on Thirteenth street, and it is probable he had a hole under it which he was trying to reach. Having an engagement to meet I was obliged to leave them. In about two hours I came back that way and turned aside to see what had become of the rat. I found the poor fellow lying within a few feet of the side-walk, his head literally torn to pieces; his eyes pecked out and blood and brains oozing from their sockets. He had been lynched in true western style, and his body left as a warning to all depredators. What crime he had committed against this community of sparrows I could not find out or imagine. I have often seen rats running around in my back yard and sparrows flying about without paying any attention to them, and this individual must have been guilty of some great outrage to bring down such punishment upon him.—WM. H. R. LYKINS, in the *K. C. Scientist.*

---

### PEPPER.

Pepper in its natural state,—that is, the kernel,—is the fruit of a plant of creeping or climbing habit and of branching growth. It attains a height of some thirty feet. Its leaves are short-stemmed, uniform and pointed. On the immense East Indian pepper plantations the young cuttings are set out in long rows and trained on poles. In this particular it bears a striking resemblance to a hop field. The plant bears fruit in its first year, but not to any great extent. It is most prolific from its fourth to its twentieth year, during which period the annual yield of a single plant is from nine to eleven pounds, on the average. The harvest season commences as soon as the uniform little green berries begin to turn red. They are then plucked and spread out on great platters, to dry in the sun's warm rays, or by means of a slow fire. This treatment causes the outer shell to shrivel and turn black. White pepper is gathered from the same plant as the black, the distinction being that the former is ground from the *ripe* berries, from which the outer black shell has first been removed. Because of this thorough maturity of the berry and the absence of the outer shell, it is much milder than the black. The strongest species of black pepper is known as the *Piper officinarum.* Its fruit, the berry, is long, having a reddish gray exterior and a very dark interior. Another, not belonging to the pepper family proper, but coming under the nightshades (*Solaneen*), is the Spanish pepper (*Capsicum longum*), whose gleaming red fruit is too familiar to require detailed mention.

---

### CURED OF A VICIOUS HABIT.

A lady had a valuable St. Bernard of excellent pedigree carefully trained and in all respects of well nigh ideal excellence, save for one fault—he would kill lambs. He was beaten and imprisoned.

While matters were in this state, a friendly farmer, who had upon some occasion got into his head the fact that the dog's mistress was fond of pets, sent her a cosset gay with ribbons and looking as innocent as innocence itself. The lady was in despair. She expected that her dog would fall upon the lamb ; but having in the past had much experience with pets she said that if this catastrophe was to happen she did not propose to have it postponed until she had become deeply attached to the new comer, and so deliberately led the lamb up to the dog, said to him that it was her lamb and directed him to watch it. The dog looked at her rather wistfully, evidently requesting permission to tear the pretty innocent, but she sternly shook her head, and, departing, left the pair together on the lawn.

When a few hours later she returned, the dog was found to have taken the lamb into his especial favor. In short, the lamb and the dog became the closest of friends, and as long as the two lived they continued to dwell together in peace and affection.

And the remarkable part of the tale is that from that day the dog ceased to molest lambs.

### BLACKHEADS, PIMPLES, ETC.

Many persons are troubled with what they suppose to be worms of the skin. These so-called worms are little masses of fat which accumulate in the obstructed oil glands of the skin. They are properly known as comedones. When one of these obstructed glands are squeezed, a little mass of fat is forced out through the opening of the gland, and thus is folded into a form which resembles that of a small grub. The small amount of dirt which has accumulated on the outer end gives it the appearance of a blackhead. This deceptive appearance has given rise to the supposition that the skin is sometimes inhabited by worms. The following mixture is recommended by a reliable medical journal as an excellent remedy for cases of this kind: Sulphuric ether, 1 oz.; carbonate ammonia, 1 dr.; boracic acid, 20 gr.; water to make 16 dr.

### A LITTLE FREE ADVERTISING.

THE most contemptible counterfeit of a man, with whom, in a varied experience of forty years, we have had the misfortune to do business on credit, is F. B. MILLS, *Seedsman, of Thorn* (now Rose) *Hill, Onondaga Co., N. Y.* We would not buy a seed or nursery tree of this fellow with any expectation of its developing into what it was sold for. How it is that such a creature thrives in a community of honorable men, is a mystery to us. *Give him a wide berth*, and patronize Vicks, of Rochester, whose word can be relied upon.

